# Transcriptome Analysis of The Inflammatory Responses of Bovine Mammary Epithelial Cells: Exploring Immunomodulatory Target Genes for Bovine Mastitis

**DOI:** 10.3390/pathogens9030200

**Published:** 2020-03-09

**Authors:** Md. Aminul Islam, Michihiro Takagi, Kohtaro Fukuyama, Ryoya Komatsu, Leonardo Albarracin, Tomonori Nochi, Yoshihito Suda, Wakako Ikeda-Ohtsubo, Victor Rutten, Willem van Eden, Julio Villena, Hisashi Aso, Haruki Kitazawa

**Affiliations:** 1 Food and Feed Immunology Group, Laboratory of Animal Products Chemistry, Graduate School of Agricultural Science, Tohoku University, Sendai 980-8572, Japan; aminul.vmed@bau.edu.bd (M.A.I.); takagimichihiro@gmail.com (M.T.); fukuyama.k.mc0511@gmail.com (K.F.); dondonassima26@gmail.com (R.K.); leonardogenius5@gmail.com (L.A.); wakako.ohtsubo.a7@tohoku.ac.jp (W.I.-O.); jcvillena@cerela.org.ar (J.V.); 2 Livestock Immunology Unit, International Education and Research Center for Food and Agricultural Immunology (CFAI), Graduate School of Agricultural Science, Tohoku University, Sendai 980-8572, Japan; 3 Department of Medicine, Faculty of Veterinary Science, Bangladesh Agricultural University, Mymensingh-2202, Bangladesh; 4 Laboratory of Immunobiotechnology, Reference Centre for Lactobacilli, (CERELA-CONICET), Tucuman 980-0845, Argentina; 5 Scientific Computing Laboratory, Computer Science Department, Faculty of Exact Sciences and Technology, National University of Tucuman, Tucuman 980-0845, Argentina; 6 Infection Immunity Unit, International Education and Research Center for Food and Agricultural Immunology (CFAI), Graduate School of Agricultural Science, Tohoku University, Sendai 980-8572, Japan; tomonori.nochi.a5@tohoku.ac.jp; 7 Cell Biology Laboratory, Graduate School of Agricultural Science, Tohoku University, Sendai 980-8572, Japan; 8 Graduate School of Food, Agriculture and Environment, Miyagi University, Sendai 980-8572, Japan; suda@myu.ac.jp; 9 Department of Infectious Diseases and Immunology, Faculty of Veterinary Medicine, Utrecht University, 3584 CL Utrecht, The Netherlands; v.rutten@uu.nl (V.R.); W.vanEden@uu.nl (W.v.E.); 10 Department of Veterinary Tropical Diseases, Faculty of Veterinary Science, University of Pretoria, Private bag X20, Hatfield 0028, South Africa

**Keywords:** bovine mammary epithelial cells, LPS, TLR2, TLR4, transcriptome, inflammation, cyclophilin A

## Abstract

Bovine mastitis is the inflammatory reaction of the mammary gland and is commonly caused by bacterial infections in high-yielding dairy cows. The detailed investigation of the immunotranscriptomic response of bovine mammary epithelial (BME) cells to pattern recognition receptors (PRRs) activation by microbial-associated molecular patterns (MAMPs) can be of great importance for understanding the innate immune defense mechanisms, and for exploring the immunomodulatory candidate genes. In this work, we investigated the transcriptome modifications of BME cells after the in vitro stimulation with *Escherichia coli* derived lipopolysaccharide (LPS) and heat-killed *Staphylococcus aureus* JE2 and *S. aureus* SA003. In addition, the effect of Pam3CSK4 (a synthetic triacylated lipopeptide that activates Toll-like receptor 2 (TLR2)), and the intracellular chemotactic protein cyclophilin A (CyPA), which is secreted by BME cells during mastitis, in the expression changes of selected cytokines and chemokines were evaluated by qPCR. Microarray analysis identified 447, 465 and 520 differentially expressed genes (DEGs) in the BME cells after LPS, *S. aureus* JE2 and *S. aureus* SA003 stimulation, respectively. A major differential response in the inflammatory gene expression was noticed between the stimulation of LPS and *S. aureus* strains. Unlike the *S. aureus* strains, LPS stimulation resulted in significant upregulation of *CCL2*, *CXCL2*, *CXCL3*, *CXCL8,* *IL1α* and *IL1β,* which were confirmed by qPCR analysis. Pam3CSK4 was not able to induce significant changes in the expression of cytokines and chemokines in challenged BME cells. The exogenous CyPA administration was able to upregulate *CXCL2*, *CXCL3*, *CXCL8, IL1α* and *IL1β* expression in BME cells indicating its ability to promote inflammation. The identification of transcriptional markers of mastitis specific for individual inflammatory factors such as LPS, Pam3CSK4 or CyPA, which can be evaluated in vitro in BME cells, may enable the development of novel diagnostics and/or immunomodulatory treatments, providing new tools for the effective management of mastitis in dairy cows. The results of this work are an advance in this regard.

## 1. Introduction

Bovine mastitis, defined as the inflammation of the mammary gland, is a frequent and contagious infectious disease of high-yielding cows. Alterations in milk composition, reduced milk quality and quantity, and increased costs for treatments, labor and culling account not only for huge economic losses but also create concerns for animal welfare and human health [1,2]. Mastitis is classified as clinical or subclinical based on the appearance of the inflammation of the udder [3]. *Escherichia coli* and *Staphylococcus aureus* are among the most prevalent Gram-negative and Gram-positive bacterial pathogens that cause mammary gland infection in dairy cows [4]. It has reported that *E. coli* infection results in clinical mastitis which is characterized by acute symptoms of inflammation in the milk collecting cistern and the teat by a reduced milk production and an elevated somatic cell count [5]. On the other hand, *S. aureus* is responsible for one-third of cases of clinical and subclinical mastitis in dairy cattle which is characterized by less severe inflammation and is sometimes asymptomatic [3]. The severity of mastitis largely depends on the patterns of interactions between invading pathogens and the bovine mammary epithelial (BME) cells [6]. Accumulated research revealed that Gram-negative bacteria provoke a strong inflammatory response through a vigorous stimulation of cytokine synthesis in the mammary gland, resulting in the activation of the local and systemic inflammatory response [5,7]. On the other hand, it was reported that Gram-positive bacteria elicit a much weaker immune reaction of the udder and generally no strong systemic immune response is detected [8,9]. Therefore, in-depth understanding of the pathogen-specific molecular mechanisms involved in the generation of mammary gland immune responses could be of great importance to explore and select effective control measures of specific pathogen-induced mastitis in dairy cows.

When pathogenic bacteria enter the udder lumen via the teat canal, they interact with BME cells in order to establish colonization. This pathogen-BME cells interaction results in the release of inflammatory mediators and chemo-attractants that recruit and stimulate immune cells which exert their antibacterial activities locally and amplify the inflammatory response [10,11]. Therefore, it is considered that BME cells stand at the frontline in the resistance against bacterial infections in mammary glands. A number of studies have shown that BME cells are able to sense bacteria or bacterial products, and that they react by up-regulating several sets of genes involved in the inflammatory response [12,13,14,15,16,17]. The innate immune response of mammary gland initiates through the recognition of microbes associated molecular patterns (MAMPs) by the patterns recognition receptor (PRRs), such as Toll-like receptors (TLRs) expressed in BME cells. The MAMPs-mediated activation of TLRs results in several downstream cell-signaling events that induce the expression of cytokine and chemokines and trigger inflammatory responses [12,13,14,15,16,17]. Although it has been demonstrated that the recognition of MAMPs by TLRs expressed in BME cells is a key event in the generation of mammary inflammation [12], detailed transcriptomic studies evaluating the response of those cells to TLRs activation has not been widely performed [18,19].

In vivo studies to uncover the mastitis-associated gene expression changes in BME cells of lactating mammary gland require the use of a large number of animals to obtain statistically robust results because these out-bred populations exhibit considerable genetic variation. Though short-term in vitro experiments using the primary cell cultures have some advantages of reflecting the appropriate mitogenic responses, isolation of primary epithelial cells from the mammary gland tissue of lactating cows is relatively difficult as compared to that of prepubertal animals [20]. On the other hand, the use of untransformed cell lines has the advantage over primary cultures in that they are able to replicate over several passages, keeping the same functional characteristics. In this work, we conducted transcriptome profiling of BME cells that were cloned from mammary tissue of a 200-day pregnant Holstein cow [21] after in vitro stimulation with either *E. coli* derived lipopolysaccharide (LPS) or heat-killed *S. aureus* JE2 or *S. aureus* SA003 strains. In addition, we compared the innate immune response triggered by LPS in BME cells with those induced by the stimulation with Pam3CSK4 (a synthetic triacylated lipopeptide that activates toll-like receptor 2 (TLR2)) and cyclophilin A (CyPA), an intracellular chemotaxis protein secreted by BME cells during inflammatory reactions which has being proposed as a marker for the early diagnosis of bovine mastitis [18,22,23,24,25]. The identification of transcriptional markers of mastitis specific for individual inflammatory factors such as LPS, Pam3CSK4 and CyPA, which can be evaluated in vitro in BME cells, may enable the development of novel diagnostics and/or immunomodulatory treatments, providing new tools for the effective management of mastitis in dairy cows.

## 2. Results

### 2.1. Expression Patterns of Toll-like Receptors in BME Cells

**Figure 1:**
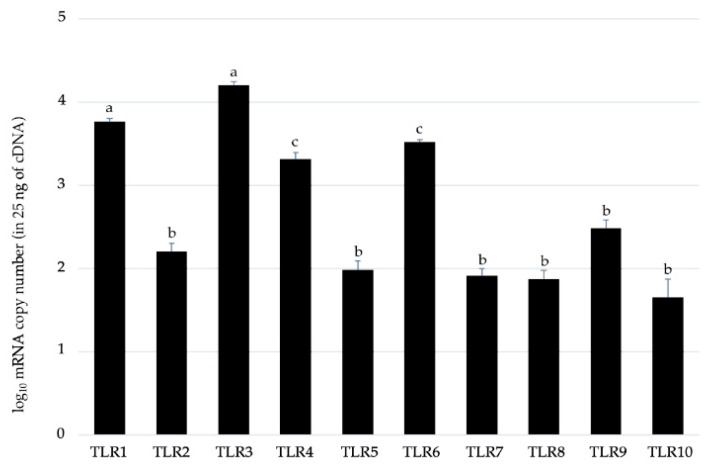
RT-qPCR-based absolute quantification of Toll-like receptors (TLRs) mRNA expression levels in a 25 ng of cDNA prepared from untreated bovine mammary epithelial (BME) cells. Results presented are as mean ± SD of four independent experiments. Different letters (a, b, c) indicates statistically significant (*p* < 0.05) difference between them.

We first aimed to evaluate the expression patterns of the members of TLR family in BME cells. The results demonstrated that the ten members of bovine TLR family are expressed in this bovine epithelial cell line (Figure 1). Among the TLR members, TLR1, TLR3, TLR4 and TLR6 were strongly expressed with a copy number greater than 10^3^. 

### 2.2. Differential Gene Expressions in BME Cells after Stimulation with LPS and Heat-killed S. auresus Strains 

A total of 1432 transcripts were differentially expressed in BME cells after stimulation with LPS or *S. aureus* JE2 or *S. aureus* SA003 (Table 1). LPS stimulation resulted in the differential regulation of 447 transcripts, of which 222 were upregulated and 207 were downregulated. Likewise, *S. aureus* JE2 stimulation as compared to the control showed 465 transcripts, with 227 and 238 transcripts upregulated and downregulated, respectively. Furthermore, *S. aureus* SA003 stimulation as compared to control showed 520 transcripts, with 226 and 294 transcripts upregulated and downregulated, respectively.

**Figure 2:**
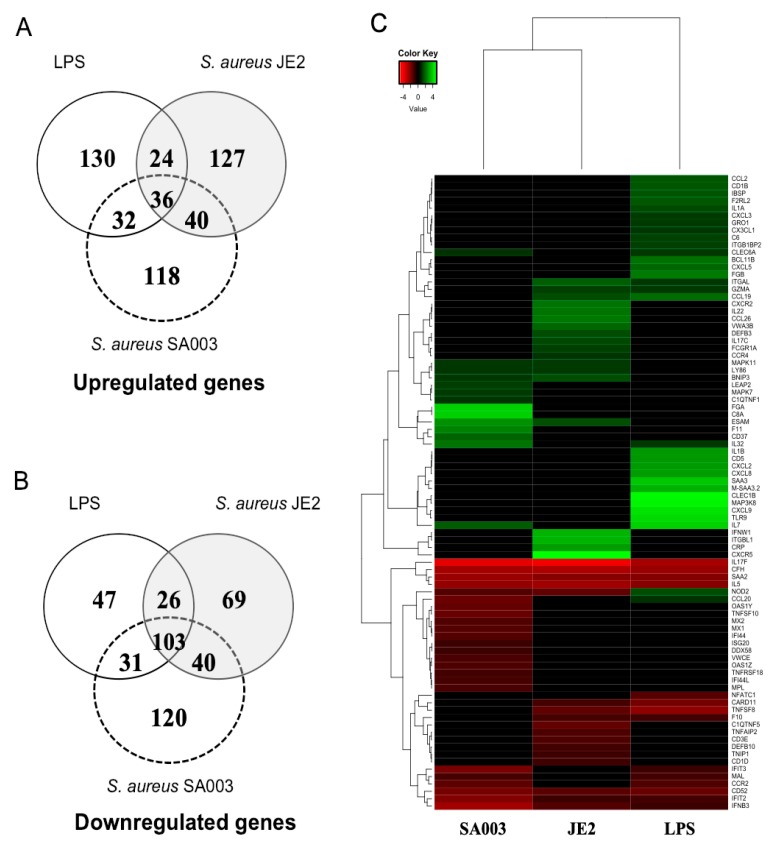
Overview of the differentially expressed genes (DEGs) in BME cells. **A.** Number of upregulated genes, **B.** Number of downregulated genes. **C.** Hierarchical clustering of DEGs.

In addition, there were 139 differentially expressed genes shared by LPS, *S. aureus* JE2 and *S. aureus* SA003 stimulations. Among them, 36 were up- and 103 were downregulated genes (Figure 2A,B). A total of 189 differentially expressed genes were common between LPS and *S. aureus* JE2 stimulation, where 60 and 129 were up- and downregulated, respectively. In addition, 100 differentially expressed genes were common between LPS and *S. aureus* SA003. A total of 202 genes were shared by *S. aureus* JE2 and *S. aureus* SA003 stimulations, of which 68 were up- and 134 were downregulated genes, respectively (Figure 2A,B). Hierarchical clustering of differentially expressed genes (DEGs) indicate that LPS and *S. aureus* stimulation are differently clustered, and the two *S. aureus* strains also differed between them (Figure 2C). The DEGs were distributed into ten distinct clusters (Figure 2C). The full list of DEGs are provided with the Supplementary files Tables S1–S3.

### 2.3. Gene Ontology (GO) Terms Enriched by DEGs 

The LPS-induced DEGs were found to be involved in the significant enrichment of GO biological terms including “Cell chemotaxis,” “Inflammatory response,” “Positive regulation of nitric-oxide synthetase biosynthesis process,” “Response to molecule of bacterial origin” and “Negative regulation of extrinsic apoptotic signaling pathway” (Table 2). The DEGs after *S. aureus* JE2 stimulation were predicted to be involved in the significant enrichment of GO biological terms, namely “Cell differentiation,” “Cell adhesion,” “Inflammatory response,” “Blood coagulation” and “Chemotaxis” while *S. aureus* SA003-induced DEGs are involved with the enrichment of GO terms including “Innate immune response,” “Cell surface receptor signaling pathway,” “Blood coagulation,” “signal transduction” and “Positive regulation of NF-kB signaling” (Table 2).

### 2.4. Differential Responses in Inflammatory Gene Gxpression in BME Cells after Stimulation with LPS and Heat-Killed and S. aureus Strains

Next, we extracted the inflammatory response related genes from the list of DEGs and compared their expression patterns between LPS and *S. aureus* stimulations. Results demonstrated that expression of *IL1α, IL1β, CCL2*, *CXCL2*, *CXCL3*, *CXCL5*, *CXCL8*, *CXCL9* and *C6* were significantly upregulated in BME cells following LPS stimulation while their expressions were not significantly modified after stimulation with either *S. aureus* strains (Table 3). The expression of *CRP* was increased after *S. aureus* stimulation while not modified after LPS stimulation. The expression of *PPIA* (also called CyPA) expression remained unchanged irrespective of stimulation (Table 3).

### 2.5. Inflammatory Gene Expression in BME Cells after LPS or Pam3CSK4 Stimulation

**Figure 3:**
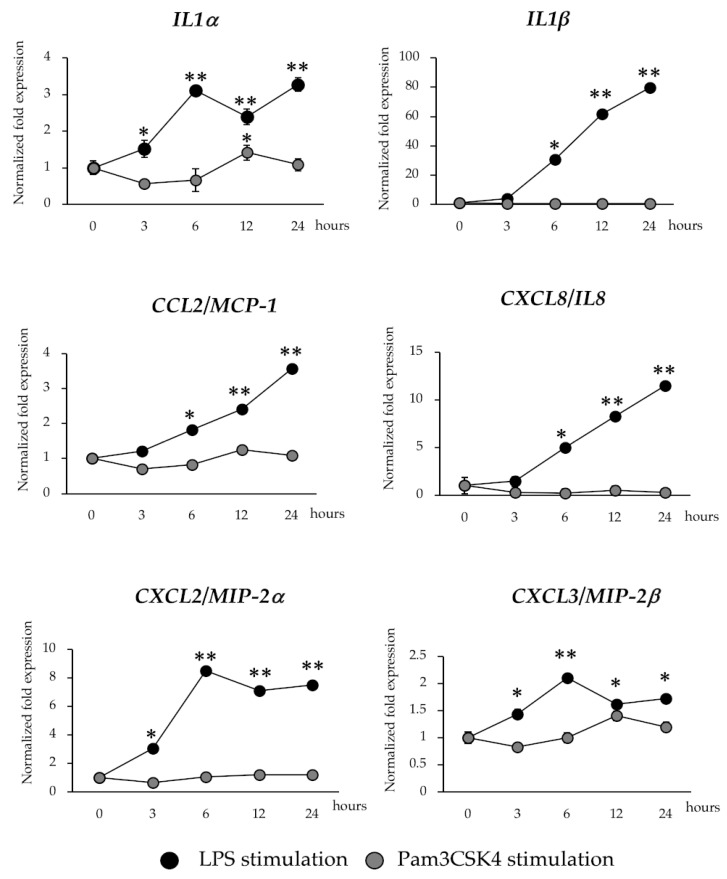
Expression patterns of inflammatory cytokines and chemokines genes in bovine mammary epithelial (BME) cells after lipopolysaccharide (LPS) and Pam3CSK4 stimulation. The expression of interleukin 1 alpha (IL1α), *IL1β*, C-C motif chemokine ligand 2 (*CCL2*), C-X-C motif chemokine ligand 2 (*CXCL2*), *CXCL3* and *CXCL8* were determined in BME cells after 3, 6, 12 and 24 hours of LPS and Pam3CSK4 stimulation by qPCR. BME cells with no LPS treatment were used as basal controls (hour 0). The results represent data (mean ± SD) from three independent experiments. Significantly different compared to basal control: *, *p* < 0.05, **p < 0.01.

In order to confirm the microarray expression results, we measured the mRNA expression dynamics of six selected inflammatory genes after the stimulation of BME cells with LPS or Pam3CSK4 by using RT-qPCR. As shown in Figure 3, the activation of TLR4 significantly increased the expression of *IL1α* and *IL1β* that was in accordance to microarray expression results. The levels of *IL1β* continuously increased until 24 hours after stimulation, while Pam3CSK4 stimulation did not induce significant variations in the expression levels of *IL1β* when compared to basal levels, showing a marked contrast with the results observed for the LPS stimulation (Figure 3). Pam3CSK4 was able to induce significant increases in the expressions of *IL1α* in BME cells at hour 12 post-stimulation. However, the increases of *IL1α* in Pam3CSK4-treated BME cells were lower than the observed for LPS-treated cells. In addition, the expressions of the chemokines *CCL2* (*MCP-1*), *CXCL8* (*IL8*), *CXCL2* (*MIP-2α*) and *CXCL3* (*MIP-2β*) were evaluated in BME cells after LPS or Pam3CSK4 stimulation (Figure 3). The mRNA levels of *CCL2* and *CXCL8* continuously increased until 24 hours after LPS stimulation while *CXCL2* and *CXCL3* reached their highest values on hour six and then they maintained similar expression levels. Pam3CSK4 did not induce significant variations in the expression levels of four chemokines evaluated when compared to basal levels (Figure 3). 

### 2.6. Inflammatory Gene Expression in BME Cells after CyPA Stimulation

**Figure 4:**
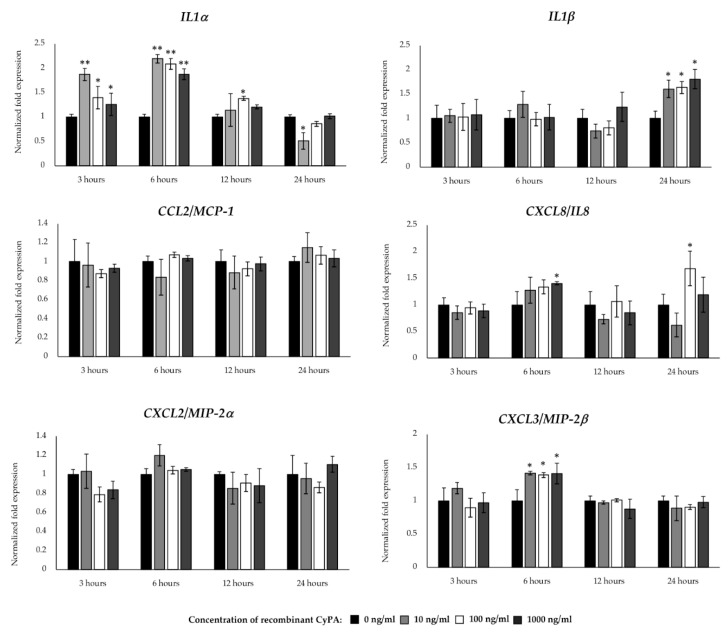
Expression patterns of inflammatory cytokines and chemokines in bovine mammary epithelial (BME) cells after recombinant bovine cyclophilin A (CyPA) stimulation. The expression of interleukin 1 alpha (IL1α), *IL1β*, C-C motif chemokine ligand 2 (*CCL2*), C-X-C motif chemokine ligand 2 (*CXCL2*), *CXCL3* and *CXCL8* were determined by qPCR in BME cells after 3, 6, 12 and 24 hours of stimulation with different concentrations of CyPA (10, 100 or 1000 ng/mL). BME cells with no CyPA treatment were used as controls (0 ng/mL). The results represent data (mean ± SD) from three independent experiments. Significantly different compared to control in the same time point: *, *p* < 0.05.

Cyclophilins, also called immunophilins, are a group of proteins having peptidyl-prolyl cis-trans isomerase activity that are secreted from mammalian cells in response to infection, cellular hypoxia or oxidative stress [22,23]. CyPA is able to promote cell signaling activation through its interaction with the intracellular receptor for the immunosuppressive drug cyclosporine A [24] or the extracellular matrix metalloproteinase inducer (EMMPRIN, also called CD147) [22]. In a recent study, we observed that extracellular CyPA possesses chemotactic activity for bovine peripheral blood cells [25], which indicate its ability to influence the inflammatory immune responses. Then, we next aimed to evaluate whether CyPA might have the ability to modulate the innate immune response of BME cells. For this purpose, BME cells were stimulated with bovine recombinant CyPA at three different doses and the expression of inflammatory cytokines and chemokines were evaluated at different time points by qPCR. As shown in Figure 4, all the doses of CyPA significantly increased the expression of *IL1α* at hours 3 and 6, as well as *IL1β* at hour 24 in BME cells when compared with unstimulated cells. In addition, all the doses of CyPA were able to significantly increase the expression of *CXCL3* at hour 6, while no variations were observed in *CCL2* and *CXCL2* (Figure 4). Significant increases of *CXCL8* were detected in BME cells at hours 6 and 24 when 1000 and 100 ng/mL of CyPA were used, respectively (Figure 4).

## 3. Discussion

The generation of inflammatory response is a vital part of the innate defenses against pathogens infecting the mammary gland mucosa. BME cells have an important role in the activation of the mammary gland innate immunity through their ability to recognize pathogens and release of inflammatory mediators that initiate the inflammatory response [10,11]. After the recognition of MAMPs by the host PRRs such as TLRs, mammary epithelial cells initiate a downstream signal transduction, which in turn lead to the activation of transcription factors including NF-kB (12). Those transcription factors modulate the expression of genes on mammary epithelial cells and induce the secretion of proinflammatory cytokines and chemokines, type I interferons, and antimicrobial peptides which coordinately induce local and systemic inflammatory responses that allows the elimination of the pathogens [12]. The results of the present study demonstrated the expression of TLR family members in BME cells, supporting the hypothesis that BME cells are capable of generating inflammatory responses against different types of pathogen-derived MAMPs through the activation of TLR pathways [12,14,26]. Moreover, by using a transcriptomic approach, we showed that BME cells are capable of modifying their gene expression patterns in response to TLR4 activation. GO enrichment of DEGs revealed that the stimulation of BME cells with LPS induced the upregulation of genes involved in cell chemotaxis, response to bacterial molecules, and inflammatory responses. 

Notably, there was a major difference in expression of inflammatory mediators in BME cells between LPS and *S. aureus* stimulations. The 12 h post LPS stimulation resulted in remarkable changes in expression of chemokines but it was not the case for infection with both *S. aureus* strains. Several in vitro studies based on exposure of BME cells to killed bacteria or purified bacterial MAMPs have shown that BME cells respond differently to *E. coli* and *S. aureus* [4,14,15,17,27,28,29]. This may explain the considerable variations in the initial recognition and subsequent pathogenesis of mastitis caused by these two pathogens. A previous study reported that severe inflammatory symptoms may be caused by the induction of *IL1* and *TNF-α* expression in *E. coli*-challenged bovine mammary gland epithelial cells, while *S. aureus* quickly triggered an increased expression of *IL6* [29]. Stimulation of BME cells with LPS for 24 h induced a marked increase in *IL1*, *CXCL6*, *CXCL8*, *TNF-α* and β-defensin expression while lipoteichoic acid (LTA, MAPM of Gram-positive bacteria) stimulation did not show any significant changes of these genes [15]. Activation of type IFN I pathway along with higher upregulation of chemokines (*CXCL10*, *CCL2*, *CCL5*, and *CCL20*) were reported in the case of LPS stimulation but not for *S. aureus* stimulation in BME cells [14]. However, the ability of *S. aureus* infection to evade the host’s immunity and to continue life-long mild inflammation [9] indicate that chemokine could be a late event in *S. aureus*/BME cells interaction, on the opposite of LPS/BME cell interaction. In this regard, it was reported the over expression of *CCL5* and *CXCL10* only after 24 h exposure of BME cells to heat killed *S. aureus* [30]. It should be noted that the present work has the limitation of testing only a single time point; therefore, a time series investigation of transcriptome shifting would provide better insight of *S. aureus* mediated inflammatory response in BME cells.

**Figure 5:**
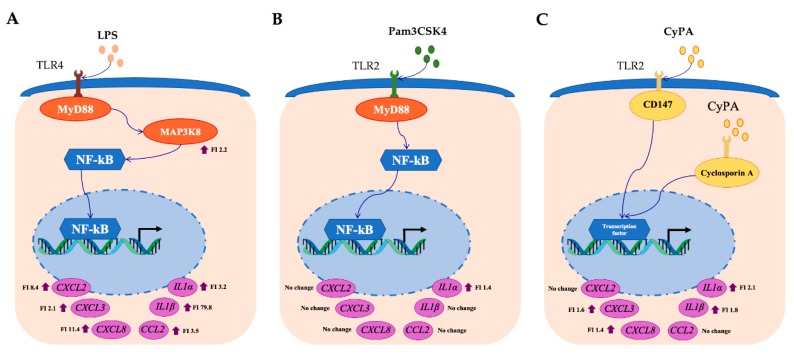
Comparative profiles of selected inflammatory gene expressions in BME cells after LPS (**A**), Pam3CSK4 (**B**) and CyPA (**C**) stimulation. The mRNA of *IL1α*, *IL1β*, *CCl2*, *CXCL8*, *CXCL2*, *CXCL3*, were upregulated after LPS stimulation. Among them only IL-1α, TLR2 and NOD2 were upregulated but others have no changes after Pam3CSK4 stimulation. While *CXCL3*, *CXCL8*, *IL1α*, and *IL1β* were upregulated and *CXCL2* and *CCL2* had no change after CyPA stimulation. FI, Fold increase.

Our transcriptomic analysis revealed that the most notable change in LPS-challenged BME cells was the significant increase in the expression of *IL1α*, *IL1β, CCL2*, *CXCL8*, *CXCL2* and *CXCL3* (Figure 5A). Those inflammatory cytokines and chemokines have been associated with protection against pathogens infecting the bovine mammary gland since bacterial growth can be efficiently inhibited through the activity of leucocytes recruited from the blood into the mammary tissue [31,32]. Earlier studies by Waller [33], evaluating the effect of a polyclonal antibody to recombinant bovine IL-1β during LPS-induced inflammation in the bovine teat cistern, demonstrated the chemotactic effects of this cytokine and its protective role in the recruitment of neutrophils into the mammary gland. Studies in primary cell cultures of BME cells obtained from high or low mastitis-resistant heifers challenged with heat-inactivated *E. coli* showed higher expression levels of *IL1β* and *IL8* in resistant animals when compared with susceptible cows [34]. Interestingly, it was reported that mammary epithelial cells constitutively secrete the chemokine CXCL3 and that normal milk contains active concentrations of this neutrophil chemoattractant protein [35]. The expression of this chemokine is increased in the course of mammary gland infections to act in synergy with other chemokines or cytokines to recruit leucocytes [36]. On the other hand, in addition of being a powerful neutrophil chemoattractant, CXCL2 shows direct antimicrobial properties against *E. coli* [37]. 

The results of the present work indicate that our in vitro BME cells system could be of value for the evaluation of immunomodulatory treatments aimed to improve the TLR4-mediated innate immune response in the bovine mammary gland and the protection against infections by Gram-negative pathogens. It should be noted that the inflammatory response in the mammary epithelium is beneficial for the clearance of pathogens and for the protection against infection; however, uncontrolled inflammation can be harmful and even fatal to the host [38,39]. The appropriate regulation of the production of proinflammatory mediators is highly important to maintain immune homeostasis and to prevent excessive inflammatory injury to the mammary gland. Thus, our in vitro BME cells system could be also of value for the selection and characterization of immunomodulatory treatments aimed to modulate the excessive inflammatory response that could become harmful for the bovine host. 

The findings of this study also indicate that our in vitro BME cells system would be efficient for the selection and study of immunomodulatory treatments aimed to improve the innate immune response against Gram-positive pathogens in the bovine mammary gland. This limitation of our system could be related to the biology of MAPMs-PRRs interactions in the bovine mammary mucosa. It has been described that the production of chemotactic factors and the infiltration of immune cells in the mammary gland differs between Gram-positive and Gram-negative pathogens [15]. Several in vitro studies based on the exposure of BME cells to viable or non-viable bacteria as well as purified bacterial MAMPs have shown that mammary epithelial cells respond differently to Gram-positive (mainly *Staphylococcus aureus*) and Gram-negative (mainly *E. coli*) pathogens [14,15,16,27,28,29]. It was reported that in experimentally induced mastitis by *E. coli* challenge, increased concentration of chemokines and cytokines were found in milk samples. However, those inflammatory mediators were undetectable or were present in low concentrations during *S. aureus* mastitis [40]. In addition, the challenge of BME cells with LPS significantly increased the expression of *TNF-α*, *IL1β* and *CXCL6* while the stimulation of these cells with Pam3CSK4 did not alter the expression of these genes [15]. Our results are in line with those previous findings since significant differential expression patterns in chemotactic factors were observed between LPS and Pam3CSK4-stimulated BME cells. We confirmed the improved expression of *IL1β* in LPS-stimulated BME cells and the lack of variation in this cytokine in Pam3CSK4-stimulated BME cells. Moreover, we extended those previous findings by demonstrating the upregulation of *IL1α*, *CCL2*, *CXCL8*, *CXCL2* and *CXCL3* in LPS-stimulated but not in Pam3CSK4-stimulated BME cells (Figure 5B). 

It should be noted that the experiments in this work were limited to the evaluation of Pam3CSK4 that signals mainly through TLR2 [41]. Several other PRRs are involved in the recognition of Gram-positive pathogens and in the generation of innate immune responses. The combination of several MAMPs from Gram-positive pathogens acting on different PRRs may explain the findings of Lee et al. [42], who described higher levels of IL-8 in cells from mammary gland quarters infected with *S. aureus* relative to those infected with *E. coli*. Therefore, more detailed transcriptomic studies evaluating the effects of different Gram-positive pathogens-derived MAMPs (lipoproteins, peptidoglycans, teichoic acids) alone or in combinations as well as viable bacteria such as *S. aureus* in BME cells would be of importance to definitively establish the value of our system for the study of the bovine mammary gland inflammatory response triggered by Gram-positive bacteria.

In addition to MAMPs from pathogens, several other factors are able to trigger and amplify inflammatory responses in the mammary gland including cytokines, chemokines or reactive oxygen species. As mentioned before, CyPA is a protein secreted from mammalian cells in response to infection or inflammation [22,23,24]. It was reported that reactive oxygen species induce CyPA secretion from vascular smooth muscle cells resulting in the expression of the endothelial cell adhesion molecule and the aberrant infiltration of inflammatory cells in the aortic wall [43,44]. In addition, our recent study with bovine peripheral blood cells in vitro treated with recombinant bovine CyPA revealed that the granulocytes migrate towards this chemotactic molecule and the migration could be inhibited by pre-treatment with an anti-bovine CyPA antibodies (25). Then, we were also interested in evaluating the ability of recombinant bovine CyPA to modulate the inflammatory response of BME cells, not only to increase our knowledge about the immunobiology of this molecule, but also to determine if our system can be used to study the inflammatory response triggered by an eukaryotic molecule not included in the MAMPs category. Our results showed that the stimulation of BME cells with CyPA resulted in an increase of inflammatory cytokines and chemokine expression (Figure 5C). In particular, the CyPA was able to significantly increase the expression of *IL1β, IL1α*, *CXCL8* and *CXCL3* in BME cells. As mentioned earlier, IL-1β has chemotactic effects inducing the recruitment of neutrophils [33]. On the other hand, it is well established that CXCL8 induces chemotaxis in target cells, primarily neutrophils but also other granulocytes, causing them to migrate toward the site of infection while CXCL3 and CXCL2 controls migration and adhesion of neutrophils and monocytes [32]. Thus, the results obtained here indicate that CyPA, in addition to having its own chemotactic properties, can enhance this function by increasing the expression of chemokines by BME cells.

## 4. Materials and Methods 

### 4.1. Cell Line 

The bovine mammary epithelial (BME) cell line used in this study was originally developed by our group and the detail protocols for establishing the cell clone are available in the previous publication [21]. BME cells were isolated from mammary gland tissue taken from a 200-days pregnant Holstein cow as per the methods described and cryopreserved in liquid nitrogen. It has been demonstrated that this BME cell clone is functionally unaffected by cryopreservation and it is responsive to mitogen and lactogenic hormones [21].

### 4.2. Growth and Maintenance of BME Cell Line

For the present study, the cryopreserved BME cells were thawed and cultured in Dulbecco’s modified Eagle medium (DMEM, Gibco, Paisley, Scotland, UK) supplemented with 20% fetal calf serum (FCS; Sigma-Aldrich, Tokyo, Japan), 100 U/mL penicillin and 100 μg/mL streptomycin (Gibco™ 15140122, Life Technologies), transferrin (5 mg/mL) and sodium acetate (5 mM) as a growth medium. For the passage, BME cells were seeded 1 × 10^4^/cm^2^ in the cell culture flask (Sumitomo Bakelite Co., Ltd., Tokyo, Japan), and incubated at 37 °C in a humidified atmosphere of 5% CO_2_. The cell culture medium was changed every 48 h and at least three consecutive passages were performed before the challenge experiments were conducted.

### 4.3. TLRs Copy Number Analysis 

For absolute quantification of TLRs family mRNA, four pools of BME cells were subjected to prepare the cDNA standards for bovine TLR 1-10. Total RNA was isolated from BME cells and cDNAs were synthesized by reverse transcription from 5 μg of total RNA using olio (dT) primers and ThermoScript RNase H-reverse transcriptase as previously described [45]. The cDNA standards were purified and quantified spectrophotometrically. TLR and β-actin cDNAs were amplified by PCR using the primers listed in Supplementary Table S4. The purified PCR products were inserted into the vector pGEM-Teasy DNA (Promega, Madison, WI, USA). We confirmed the homology of each insert with dideoxy chain termination method using a DNA sequencer (4000 L; Li-Cor, Lincoln, NE, USA) and the SequiTherm EXCEL™ II DNA Sequencing Kit-LC (Epicentre Biotechnologies, USA). The copy number for each mRNA was determined using the following formula: Copy number (copies) = 6.02 × 10^23^ (copies/mol) × measurement (g)/MW (g/mol). MW = size in bp × 660 (g/mol/bp). Aliquots of standard cDNA containing 10^7^–10^2^ pg/μl were created for TLRs and used as assay standards.

### 4.4. Preparations of Stimulants

Two staphylococcal strains: *S. aureus* JE2 and *S. aureus* SA003 were grown in the antibiotic-free α-minimum essential medium. The bacteria were propagated up to mid-logarithmic phase (OD_620_ = 0.4) and washed twice with phosphate buffered saline (PBS). The determination of bacterial density was made by limiting dilution of washed bacteria using the SLGC bacterial calculator (Sansho Co. Ltd., Tokyo, Japan) according to the manufacturer’s instructions. Bacteria were resuspended in PBS, heat-killed by incubation at 95 °C for 20 minutes and stored at −20 °C until use. For mimicking the *E. coli* infection, we used commercially available lipopolysaccharide derived from *Escherichia coli* 55: B5(L6529-1mg, Sigma-Aldrich, Tokyo, Japan). In addition, we used commercially available Pam3CSK4 (Nacalai Tesque, Inc., Kyoto, Japan) for demonstrating TLR2 mediated inflammation.

### 4.5. Experimental Challenge to BME Cells and Sampling for Gene Expression Study

Five days-confluent BME cells (5 × 10^5^ cells/well) were seeded in a twelve-well cell culture plate. After settling down, three pool of adherent cells were stimulated with either LPS (1.0 µg/mL) or heat-killed bacteria of *S. aureus* JE2 (5 × 10^7^ cells/well) or *S. aureus* SA003 (5 × 10^7^ cells/well) strains or kept unstimulated as control. The cultures were maintained at 37 °C in a humidified atmosphere of 5% CO_2_ for 12 h and then harvested for RNA extraction. The microarray-based gene expression analyses was performed in a single replicate for each treatment, followed by the RT-qPCR validation in three biological replicate samples as described below.

In a second set of experiments, BME cells (5 × 10^5^ cells/well) were plated in a twelve-well cell culture plate and stimulated with either LPS (1.0 µg/mL) or Pam3CSK4 (100 ng/mL) or recombinant bovine CyPA (10, 100 or 1000 ng/mL) or kept unstimulated as control. The cultures were maintained at 37 °C in a humidified atmosphere of 5% CO_2_ for 3, 6, 12 or 24 h and then harvested at every time point for RNA extraction and RT-qPCR analysis as described below.

### 4.6. RNA Isolation, Labeling and Microarray Hybridization

Total RNA was extracted from the treated and untreated control cells using PureLink RNA Mini Kit (Life Technology INC., USA) along with on-column DNase treatment. RNA integrity, quality and quantity were evaluated with microcapillary electrophoresis (2100 Bioanalyzer (Agilent Technologies, Santa Clara, CA, USA) using the Agilent RNA 6000 Nano Kit (Agilent Technologies, Santa Clara, CA, USA). Samples with RNA integrity number (RIN) greater than 9 were used for microarray study. 

Microarray analyses were performed using a one-color microarray-based Gene Expression Analysis Kit (Agilent, USA) and Bovine (V2) Gene Expression Microarray 4 × 44K oligonucleotide slides (Agilent, USA). Cyanine-3 (Cy3) labeled cRNA was prepared from 200 ng RNA using the One-Color Low Input Quick Amp Labeling Kit (Agilent) according to the manufacturer’s instructions. Dye incorporation and cRNA yield were checked with the NanoDrop ND-1000 Spectrophotometer. The Gene Expression Hybridization kit (Agilent Technologies) was used for hybridization. In brief, 1650 ng of Cy3-labelled cRNA (specific activity >10.0 pmol Cy3/ug cRNA) was fragmented at 60 °C for 30 minutes in a reaction volume of 250 mL containing 1x Agilent fragmentation buffer and 2x Agilent blocking agent following the manufacturer’s instructions. On completion of the fragmentation reaction, 250 mL of 2x Agilent hybridization buffer was added to the fragmentation mixture and hybridized to Agilent Bovine (V2) Gene Expression Microarray, 4 × 44K (G2519F) for 17 hours at 65 °C in a rotating Agilent hybridization oven. After hybridization, microarrays were washed 1 minute at room temperature with GE Wash Buffer 1 (Agilent) and 1 minute with 37 °C GE Wash buffer 2 (Agilent), then dried immediately by brief centrifugation.

### 4.7. Statistical Analysis of Microarray Data 

The normalization and differential expression analysis of microarray data were performed with GeneSpring GX software (v13.1, Agilent Technologies, USA). The log_2_ transformed expression values of probes were normalized based on 75 percentile shifts. For identification of differentially expressed genes (DEGs) following each stimulation, we compared gene expression profiles between control and each treatment: LPS, *S. aureus* JE2 and *S. aureus* SA003. In order to determine the differential expression of genes, an unpaired t-test was performed between untreated control and stimulated samples. Benjamini and Hochberg (B-H) adjustment method was applied for multiple test correction. Significant differentially expressed genes were selected on the basis of two criteria: an adjusted *p*-value (FDR, false discover rate) of less than 0.05 and a cutoff in fold change of at least 1.5. The annotation and conversion of DEGs into human orthologous ensembl gene ID were performed by using the bioDBnet tool [46] and BovineMine [47], which were subjected for downstream functional analysis. The MIMAE (minimum information about a microarray experiment) standard raw microarray dataset have been submitted to the NCBI-GEO database under the access number GSE139612.

### 4.8. Gene Ontology Analyses 

For biological interpretation of differential gene expressions, gene ontology (GO) enrichment analysis was performed using the InnateDB online tool (v5.4, [48]). The ensemble gene ID were uploaded to the InnateDB web portal and performed GO analysis. For each case, the over representation analysis (ORA) was performed using hypergeometric algorithm with Benjamini-Hochberg (B-H) multiple test correction method. 

### 4.9. RT-qPCR Validation

Quantitative two-step real-time PCR was performed to confirm the microarray results and to evaluate the effect of Pam3CSK4 and CyPA by quantifying the mRNA expression levels of six selected genes in BME cells. Primer sequences are presented in Supplementary Table S4. Total RNA was isolated from each sample using TRIzol reagent (Invitrogen, Carlsbad, CA, USA) followed by treated with gDNA Wipeout Buffer (Qiagen, Tokyo, Japan). All cDNAs were synthesized using the Quantitect reverse transcription Kit (Qiagen, Tokyo, Japan) according to the manufacturer’s recommendations. 

The RT-qPCR was performed using 7300 real-time PCR system (Applied Biosystems, Warrington, UK) using the TaqMan^®^ gene expression assay kit (Life Technologies) and TaqMan^®^ Universal Master Mix II, with UNG (Applied Biosystems, Warrington, UK). The PCR cycling conditions were 2 min at 50 °C, followed by 10 min at 95 °C, and then 40 cycles of 15 s at 95 °C, 1 min at 60 °C. The reaction mixtures contained 2.5 μL of sample cDNA, 1 μL gene expression assay and 10 μL TaqMan^®^ Universal Master mix II, with UNG, and 6.5 μL distilled water. According to the minimum information for publication of quantitative real-time PCR experiments guidelines, β-actin was used as a house-keeping gene because of its high stability across various bovine tissues [49]. Relative index was calculated as the ratio of target mRNA expression to β-actin. Then, raw data were transferred from the mean Ct values of replicated samples to copy number of the established standard curve. The normality of the log_2_ transformed data distribution were checked by Kolmogorov-Smirnov test. Comparisons between mean values were carried out using one-way ANOVA and Fisher’s least significant difference test. For every case, *p* < 0.05 was considered statistically significant. 

## 5. Conclusions

We herein presented the transcriptome profile associated with the inflammatory response of BME cells following in vitro stimulation with LPS or heat-killed *S. aureus* JE2 or *S. aureus*SA003. The challenge of BME cells with LPS resulted in the activation of the TLR4 signaling pathway that conducted to a strong inflammatory response compared with the responses induced by *S. aureus* strains or other inflammatory factors such as Pam3CSK4 or CyPA. The genes with marked variations in response to TLR4 activation in BME cells identified in the present study including *IL1β, IL1α*, *CCL2*, *CXCL8*, *CXCL2* and *CXCL3* could be used as potential biomarkers for the selection of effective immunomodulatory treatments for improving the outcome of mastitis through the use of the BME cells in vitro evaluation system. Precise studies of the transcriptional changes induced by the activation of PRRs by pathogens in BME cells are necessary to provide the scientific basis for the generation of immunointervention tools that improve the outcome of infections and protect against inflammatory damage in the mammary gland of the bovine host. The results of this work are an advance in this regard.

## Figures and Tables

**Table 1:** The number of differentially expressed genes (DEGs) in BME cells after stimulation with lipopolysaccharide (LPS) or heat-killed *S. aureus* strains. Statistically significant DEGs were detected based on the criteria of *p*-value < 0.05, false discovery rate (FDR) of 0.1 and fold change (FC) 1.5, as compared to the untreated control.

	Control vs. LPS	Control vs. *S. aureus* JE2	Control vs. *S. aureus* SA003
Upregulated	222	227	226
Downregulated	207	238	294
Total	447	465	520

**Table 2:** Gene ontology (GO) terms enriched after stimulation with LPS or heat-killed *S. aureus* strains to BME cells.

GO Terms and IDs	Genes Involved	*p*-Value
LPS stimulation:		
Cell chemotaxis [GO:0060326]	*AGTR1, CCL2, CXCL2, CXCL3, CXCL5, CXCL8, CXCL9*	0.0001
Inflammatory response [GO:0006954]	*CCL20, CCL2, CXCL2, CXCL8, CXCL9, GGT5, IL1* *α, IL1* *β*	0.002
Positive regulation of nitric-oxide synthetase production [GO:0051770]	*CCL20, CCL2, NOD2*	0.0002
Response molecules of bacterial origin [GO:0002237]	*CXCL2, CXCL8*	0.003
Negative regulation of extrinsic apoptotic signaling pathway [GO:2001240]	*IL1* *α, IL1* *β, IL17, UNC5B*	0.006
*S. aureus* JE2 stimulation:		
Cell differentiation [GO:0030154]	*CCDC135, DLL1, HEMGN, MGP, NEUROD6, NKX2-2, PDX1, PIWIL3, SEMA4G, TCF23*	0.001
Cell adhesion [GO:0007155]	*ABL1, CLDN11, ITGBL1, MAG, MPDZ, NCAN, PRKCE*	0.02
Inflammatory response [GO:0006954]	*CCL26*, *CNR2*, *CRP*, *CXCR2*, *IL22*	0.04
Blood coagulation [GO:0007596]	*ABL1*, *EFEMP2*, *ESAM*, *KIF2B*, *MAG*, *PRKCE*, *PRKG2*, *SERPINB2*	0.08
Chemotaxis [GO:0006935]	*CCL26, CXCR2, CXCR5*	0.19
*S. aureus* SA003 stimulation:		
Innate immune response [GO:0045087]	*AHSG*, *APOA1*, *C8A*, *F11*, *FREM1*, *KLRD1*, *MAFB*, *MAPK10*, *OTUB1*, *PRKCE*, *SFTPA1*	0.01
Cell surface receptor signaling pathway [GO:0007166]	*GPR97*, *KLRD1*, *LEPR*, *LIFR*	0.03
Blood coagulation [GO:0007596]	*APOA1*, *ESAM*, *F11*, *KIF3C*, *PRKCE*, *TRPC3*	0.03
Signal transduction [GO:0007165]	*CAPN3, ECM1, GDF3, MAPK10, NPAS2, PDE4B, PRKCE, SYNGAP1*	0.04
Positive regulation of NF-kB signaling [GO:0043123]	*ECM1, HTR2B, PRKCE*	0.06

**Table 3:** Expression patterns of selected inflammation related genes in BME cells after stimulation with LPS or heat-killed *S. aureus* strains.

Gene Symbol	Gene Name	LPS	*S. aureus* JE2	*S. aureus* SA003
IL1α	Interleukin 1 Alpha	1.463	N.M	N.M
IL1β	Interleukin 1 Beta	2.94	N.M	N.M
CCL2	C-C Motif Chemokine Ligand 2 (also called monocyte chemoattractant protein -1, MCP-1)	1.682	N.M	N.M
CX3CL1	C-X3-C Motif Chemokine Ligand 1	1.146	N.M	N.M
CXCL2	C-X-C Motif Chemokine Ligand 2	2.953	N.M	N.M
CXCL3	C-X-C Motif Chemokine Ligand 3	1.194	N.M	N.M
CXCL5	C-X-C Motif Chemokine Ligand 5	2.007	N.M	N.M
CXCL8	C-X-C Motif Chemokine Ligand 8 (also called Interleukin 8, IL8)	3.013	N.M	N.M
CXCL9	C-X-C Motif Chemokine Ligand 9	4.379	N.M	N.M
C6	Complement component 6	1.313	N.M	N.M
CRP	C-Reactive Protein	N.M	3.012	N.M
M-SAA3.2	Mammary Serum Amyloid A3.2	3.413	N.M	N.M
SAA3	Serum Amyloid A3	3.869	N.M	N.M
PPIA	Peptidylprolyl Isomerase A (also called Cyclophilin A, CyPA)	N.M	N.M	N.M

Values shown as the log_2_ fold-change of corresponding genes, N.M., Not modified (<−1.5 to > 1.5).

## Supplementary Materials

The following are available online at https://www.mdpi.com/2076-0817/9/3/200/s1. There are four additional files provided within a zip folder. Table S1: List of DEGs after LPS stimulation to BME cells. Table S2: List of DEGs after *Staphylococcus aureus* JE2 stimulation to BME cells Table S3: List of DEGs after *Staphylococcus aureus*SA003 stimulation to BME cells. Table S4: The primer sequences used for qPCR study.
