# Supplementary material for: Transcriptome Analysis of The Inflammatory Responses of Bovine Mammary Epithelial Cells: Exploring Immunomodulatory Target Genes for Bovine Mastitis
Source: Pathogens. 2020 Mar 9;9(3):200. doi: 10.3390/pathogens9030200 (PMC7157600; doi:10.3390/pathogens9030200)
Supplement: Supplementary file 1 [file pathogens-09-00200-s001.zip › Supplementary materials/Table S4.Primer sequence used for qPCR.docx]

**Table S4**. Sequence of the primer sets used for expression study by RT-qPCR

| **Gene symbol** | **Sequence (3` to 5`) *** | **Amplicon size** | **Accession number** |
| --- | --- | --- | --- |
| ACTB | F: TGG ATT GGC GGC TCC AT  R: GCT GAT CCA CAT CTG CTG GAA | 57 | NM_173979.3 |
| IL1α | F: CAG TTG CCC ATC CAA AGT TGT T  R: TGC CAT GTG CAC CAA TTT TT | 59 | NM_174092.1 |
| IL1β | F: GAG CCT GTC ATC TTC GAA ACG  R: GCA CGG GTG CGT CAC A | 55 | NM_174093.1 |
| CCL2 | F: CAC CAG CAG CAA GTG TCC TAA A  R: CAC ATA ACT CCT TGC CCA GGA T | 65 | NM_174006.2 |
| CXCL2 | F: CTA GGC CAG CTC TAA CTG AC  R: TGG TGA TTC CTC TTT TCC CT | 107 | NM_174299.3 |
| CXCL3 | F: GAC AGT TCC TGA AAA GTG GT  R: ATA GTC CAG CAC ATC AAG TC | 104 | NM_001046513.2 |
| CXCL8 | F: TGC TCT CTT GGC AGC TTT CC  R: TCT TGA CAG AAC TGC AGC TTC AC | 61 | NM_173925.2 |
| TLR1 | F: CAT TCC TAG CAG CTA CCA CAA GCT  R: TGG GCC ATT CCA AAT AAG TTC T | 65 | NM_001046504.1 |
| TLR2 | F: GGG TGC TGT GTC ACC GTT TC  R: GCC ACG CCC ACA TCA TCT | 57 | NM_174197.2 |
| TLR3 | F: GGG CAC CTG GAG GTC CTT  R: TTC CTG GCC TGT GAG TTC TTG | 63 | NM_001008664.1 |
| TLR4 | F: AGC ACC TAT GAT GCC TTT GTC A  R: GTT CAT TCC GCA CCC AGT CT | 61 | NM_174198.6 |
| TLR5 | F: GTC CCC AAC ACC ACC AAG AG  R: GCG GTT GTG ACT GTC CTG ATA TAG | 62 | NM_001040501.1 |
| TLR6 | F: TTT ACC CTC AAC CAC GTG GAA  R: GGG CCA AAG GAA CTG AAA AAC | 66 | NM_001001159.1 |
| TLR7 | F: CAC CAA CCT TAC CCT CAC CAT T  R: GTC CAG CCG GTG AAA GGA | 67 | NM_001033761.1 |
| TLR8 | F: TGT GTT TAG AGG AAA GGG ATT GG  R: TCT GCA TGA GGT TGT CGA TGA | 60 | NM_001033937.1 |
| TLR9 | F: CAG TGG CCA GGG TAG TTT CTG  R: CCG GTT ATA GAA GTG ACG GTT GT | 73 | NM_183081.1 |
| TLR10 | F: TCT ACT GCA TCC CTA CCA GAT ATC C  R: GGG CCA TTC CAA GTA TGC TTT | 71 | NM_001076918.2 |

*F, Forward; R, Reverse
